# Contribution of Endocannabinoids to Intrinsic Motivation for Undirected Singing in Adult Zebra Finches

**DOI:** 10.3389/fphys.2022.882176

**Published:** 2022-04-12

**Authors:** Yunbok Kim, Satoshi Kojima

**Affiliations:** Sensory and Motor Systems Research Group, Korea Brain Research Institute, Daegu, South Korea

**Keywords:** endocannabinoid, motivation, songbird, dopamine, reward, vocalization, voluntary behavior, vocal learning

## Abstract

Songbirds, such as zebra finches, spontaneously produce many song renditions for vocal practice even in the absence of apparent recipients throughout their lives. Such “undirected singing” is driven by intrinsic motivation, which arises within individuals for internal satisfaction without immediate external rewards. Intrinsic motivation for undirected singing in adult zebra finches was previously demonstrated to be critically regulated by dopamine through D2 receptors. Here, we further investigate the mechanisms of intrinsic motivation for undirected singing by focusing on endocannabinoids, which modulate dopamine signaling and contribute to motivation and reward in mammals. In songbirds, endocannabinoids have been shown to be involved in the production of undirected songs, but whether they are involved in the intrinsic motivation for undirected singing remains unknown. Using latencies of the first song production following temporary singing suppression as a measure of intrinsic motivation for undirected singing, we demonstrate that systemic administration of the direct cannabinoid agonist WIN55212-2 decreases intrinsic motivation for singing and that those effects are largely reversed by the cannabinoid antagonist SR141716A co-administered with WIN55212-2. Administration of SR141716A alone or that of two indirect cannabinoid agonists did not significantly affect intrinsic singing motivation. These results suggest that endocannabinoids are critically involved in regulating intrinsic motivation for undirected singing and provide new insights into the neural mechanisms of intrinsically motivated motor behaviors.

## Introduction

**FIGURE 1:**
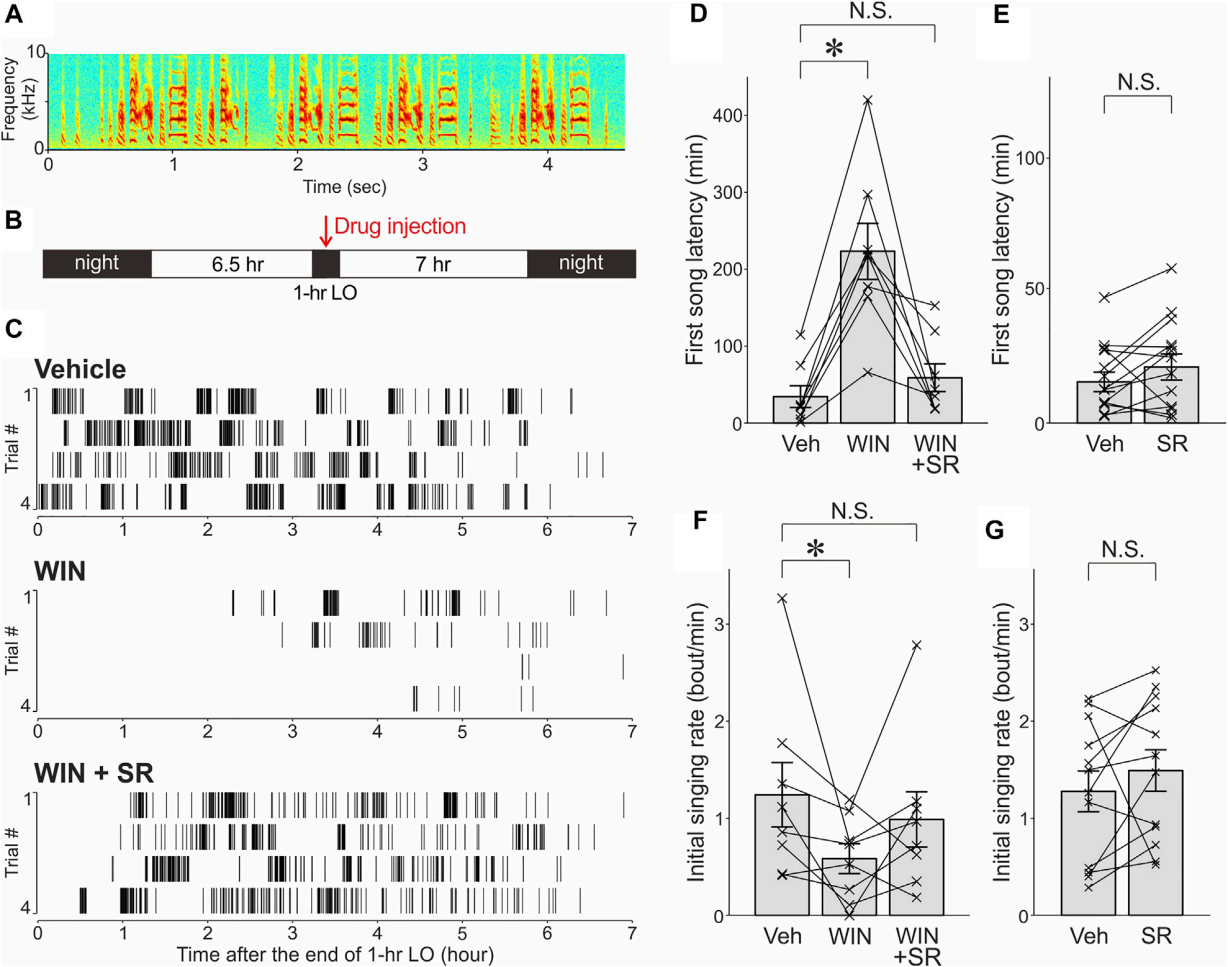
The cannabinoid agonist WIN significantly decreased intrinsic motivation for undirected singing and this effect was reversed by the cannabinoid antagonist SR. **(A)** *.* Spectrogram of an undirected song bout in a representative bird. **(B)**. Schedule of lights-out (LO) and drug administration. Drugs or their vehicles were injected 30 min prior to the offset of 1-h LO periods. **(C)**. Raster plot of song bouts produced after LO periods with vehicle (*top*), WIN (*middle*), or WIN & SR (*bottom*) administrations in a representative bird. **(D)**. The effects of WIN and SR administrations on first song latencies. First song latencies were significantly increased after WIN administrations compared with those after vehicle administrations (“Veh”), and these effects were largely reversed by co-administration of SR and WIN (mean ± SEM, *p < 0.01, significance level α was corrected from 0.05 to 0.0167). Each line indicates a single bird. N.S., not significant. **(E)**. Effects of SR administration alone on first song latencies. **(F)**. Effects of WIN and SR administrations on initial singing rates. *p < 0.05. Conventions are as in *D*. **(G)**. Effects of SR administration alone on initial singing rates.

Animals, including humans, spontaneously exhibit various behaviors, even without receiving any immediate external reward such as food or money. Such voluntary behaviors are driven by intrinsic motivation and are critical for the development and optimization of cognitive, social, and physical functions throughout life (Parisi et al., 2019; Ryan and Deci, 2000). Songbirds, such as zebra finches, offer a unique opportunity to study the neural substrates of intrinsic motivation because they spontaneously produce hundreds of renditions of stereotyped songs every day, even in the absence of apparent recipients (“undirected singing”) (Figure 1A) (Dunn and Zann, 1996; Sossinka and Böhner, 1980). Undirected singing is thought to serve, at least in part, as a vocal practice by which birds develop and optimize song structure to prepare for future courtship activity (Brainard and Doupe, 2001; Konishi, 1965; Lombardino and Nottebohm, 2000; Tumer and Brainard, 2007); other functions of undirected singing have also been suggested (EensHausberger et al., 1995; EensHausberger et al., 1997; Jesse and Riebel, 2012).

We have previously demonstrated that the intrinsic motivation for undirected singing in adult zebra finches is critically regulated by dopamine through D2 receptors (Kim et al., 2021). In mammals, dopamine signaling is modulated by endocannabinoids (El Khoury et al., 2012; Wenzel and Cheer, 2018) and they play a critical role in motivation for voluntary behaviors, such as social play and physical exercise (Muguruza et al., 2019; Parsons and Hurd, 2015; Vanderschuren et al., 2016). In songbirds, endocannabinoids are involved in undirected singing: the amount of undirected singing is dramatically decreased by systemic administration of the direct CB_1_ cannabinoid agonist WIN55212-2 (WIN), and such effects are partially reversed by the CB_1_ cannabinoid antagonist SR141716A (SR) (Soderstrom and Johnson, 2001). Although these results suggest that endocannabinoids play a role in the production of undirected songs, they do not necessarily indicate that endocannabinoids are directly involved in the intrinsic motivation for undirected singing. There is evidence that undirected singing acts as internal reward and is associated with a positive affective state (a state of “liking”) (Riters and Stevenson, 2012). Because such singing-associated reward may facilitate subsequent song production, the inhibitory effect of WIN on undirected song production could result from a reduction in singing-associated reward rather than a direct influence of the drug on intrinsic singing motivation. In support of this view, significant correlations between singing-associated reward and endocannabinoid-related gene expression have been found in songbirds (Hahn et al., 2017). Thus, it remains to be determined whether the endocannabinoid system is involved in intrinsic singing motivation (a state of “wanting”) and/or singing-associated reward (a state of “liking”).

To better understand the role of endocannabinoids in undirected singing, we examined whether they are critically involved in intrinsic singing motivation. To quantify the motivation for undirected singing, we used the latency to the first song following the temporary suppression of singing (referred to as “first song latency”), a measure recently developed and described in our previous work (Kim et al., 2021). Unlike the singing rate and song amount, the first song latency is not directly influenced by any process following the act of singing, allowing for examination of intrinsic singing motivation independent of singing-associated reward. We systemically administered WIN and SR as in a previous study that examined their effects on undirected song production (Soderstrom and Johnson, 2001), and examined their effects on the first song latency to determine the role of the endocannabinoid system in intrinsic singing motivation. We additionally examined the effects of these drugs on the mean singing rates measured over a 30-min period starting at the first song after singing suppression (“initial singing rate”), which can reflect both intrinsic singing motivation and singing-associated reward (Kim et al., 2021). Moreover, we assessed the effects of two indirect cannabinoid agonists, URB597 and VDM11, on undirected singing, both of which have been shown to enhance intrinsically motivated social play in adolescent rats (Manduca et al., 2014; Trezza and Vanderschuren, 2009, 2008a, 2008b).

## Materials and Methods

### Subjects

All the subjects were adult male zebra finches (*Taeniopygia guttata*, 90–146 days post hatching). Birds were raised in our colony with their parents and siblings until ∼60 days old and then housed with their siblings and/or other male conspeciﬁcs until the experiments started. The care and treatment of animals were reviewed and approved by the Institutional Animal Care and Use Committee (IACUC) of the Korea Brain Research Institute.

### Song Recording and Measuring Singing Motivation

Birds were housed individually in sound-attenuating chambers (MC-050, Muromachi Kikai) under a 14:10 h light:dark cycle throughout the experiments. Undirected songs, which the birds spontaneously produced in a solo context, were recorded as previously reported (Kim et al., 2021). Briefly, the output from a microphone (PRO35, Audio-Technica) positioned above the cage was ampliﬁed using a mixer (402-VLZ4, Mackie) and digitized via an audio interface (Octa-Capture UA-1010, Roland) at 44.1 kHz (16-bit). Recording was controlled by a custom-written song recording program (R. O. Tachibana at the University of Tokyo), which triggered recording if it detected four or five consecutive sound notes, each of which was defined based on the sound magnitude, duration, and intervening gap duration. Each recording ended when the silent period lasted longer than 0.5 s. Birds with sufficient singing rates (>300 song bouts per day) were used in further experiments.

Intrinsic motivation for undirected singing was quantified by measuring the latency of the first song produced following the temporal suppression of singing (“first song latency”) (Kim et al., 2021). Singing was suppressed by turning off the light in the sound-attenuating chambers for 1 or 2 h depending on the drugs administered (see below), and the time interval from the offset of the lights-out (LO) period to the onset of the first song was measured. Furthermore, the mean singing rates over a 30 min period starting at the first song following LO periods (“initial singing rates”) were measured. To obtain all songs produced during those periods, all sound files recorded during those periods were screened to exclude non-song files (which include calls and/or noise) using a previously reported semi-automated method (Kim et al., 2021).

### Drug Administrations

For pharmacological manipulation of cannabinoid signaling, the following drugs or the corresponding vehicle were injected into the pectoral muscle: the direct CB_1_ cannabinoid agonist WIN (Sigma-Aldrich, W102; 1 mg/kg); selective CB_1_ cannabinoid inverse agonist/antagonist SR (Sigma-Aldrich, SML0800; 5 mg/kg); and the indirect endocannabinoid agonists URB597 (Sigma-Aldrich, U4133; 0.2 and 1 mg/kg) and VDM11 (Sigma-Aldrich, V3264; 1 and 5 mg/kg). The doses were selected based on previous studies (Achterberg et al., 2016; Gilbert and Soderstrom, 2013; Muguruza et al., 2019; Trezza and Vanderschuren, 2009, 2008a). All drugs were stored as stock solutions in DMSO at −20 °C; WIN and VDM11 were dissolved in saline before use; SR and URB597 were dissolved in 5% Tween-80/5% polyethylene glycol/saline. All birds received a single injection of a drug or vehicle every 1–2 days with a fixed LO schedule: For all drugs except URB597, birds received 1 h LO in the middle of the day (Figure 1B) and the drug or vehicle was injected 30 min before the end of the LO periods; for URB597, birds received 2 h LO period and the drug or vehicle was injected at the beginning of those LO periods, following a previous study (Achterberg et al., 2016). Different sets of birds were used for different experiments: 8 birds were injected with WIN, the mixture of WIN and SR, and the vehicle; 13 birds were injected with SR and the vehicle; 12 birds were injected with 0.2 and 1 mg/kg of URB597 and the vehicle; 12 birds were injected with 1 and 5 mg/kg of VDM11 and the vehicle. The drugs and corresponding vehicle were injected sequentially (only a single injection per day), and those injections were repeated 2–5 times; the results (first song latency and initial singing rate) for the same drug/vehicle were averaged across injections. The temporal order of drug and vehicle injections was as follows: For the WIN and WIN + SR experiment, vehicle, WIN, and WIN + SR were injected in order but the order of WIN and WIN + SR was switched after every round of injections. For the SR alone experiment, vehicle and SR were injected alternately. For the URB597 and VDM11 experiments, vehicle, lower dose and higher dose of the drug were injected in order. We confirmed that all birds produced substantial amounts (>50 renditions) of undirected songs during the light period before each drug injection to ensure that the drug injection on the preceding day did not severely affect singing behavior on the next day.

### Statistical Analysis

To examine the effects of drug administration, bird behaviors (first song latencies and initial singing rates) were compared between those occurring after drug administration and those after vehicle administration, using a Wilcoxon signed-rank test. For multiple comparisons, the significance threshold (alpha) for rejecting the null hypothesis was adjusted using the Bonferroni correction. All statistical analyses were performed using the MATLAB software.

## Results

### The Effects of WIN and SR

Systemic administration of the cannabinoid agonist WIN at a dose that has been shown to effectively suppress undirected song production (1 mg/kg) (Soderstrom and Johnson, 2001) markedly prolonged first song latencies following temporal suppression of spontaneous undirected singing: First song latencies observed after WIN administrations were significantly longer than those after vehicle administration (Figures 1C,D; n = 8 birds, *p* = 0.0078, Wilcoxon signed-rank test with a Bonferroni correction for multiple comparisons, significance level α was corrected from 0.05 to 0.0167). Moreover, this prolongation was largely reversed by the cannabinoid antagonist SR (5 mg/kg), which was co-administered with WIN (Figures 1C,D; n = 8 birds, *p* = 0.20 vs. vehicle). The effect of SR administration alone was assessed on first song latencies in a separate experiment, and no significant effects were observed (Figure 1E; n = 13 birds, *p* = 0.17, Wilcoxon signed-rank test).

The effects of these drugs on initial singing rates were also examined. Consistent with the results of the first song latencies, the initial singing rates were significantly (but marginally) reduced by WIN administration (Figure 1F; n = 8 birds, *p* = 0.0156), and these effects were substantially reversed by SR co-administered with WIN (Figures 1C,F, n = 8 birds, *p* = 0.5 vs. vehicle). In addition, no significant effects were observed after SR administration alone (Figure 1G; n = 13 birds, *p* = 0.15).

### The Effects of URB597 and VDM11

**FIGURE 2:**
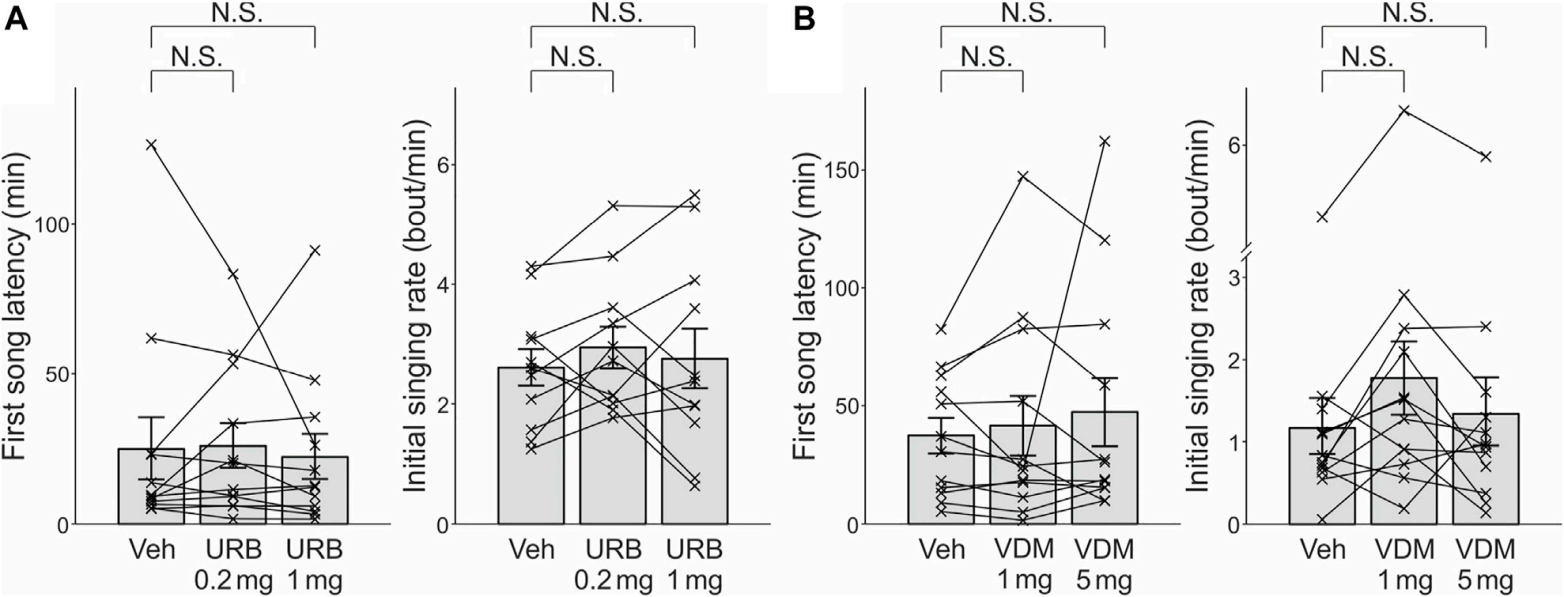
The effects of the indirect cannabinoid agonists URB597 and VDM11 on first song latencies and initial singing rates. **(A)**. URB597 administered at 0.2 and 1 mg/kg did not have significant effects either on first song latency (*left*) or initial singing rate (*right*). Conventions are as in Figure 1D *.* **(B)** *.* VDM11 administered at 1 and 5 mg/kg did not have significant effects either on first song latency (*left*) or initial singing rate (*right*).

We also examined the effects of the indirect cannabinoid agonists URB597 and VDM11 on both first song latencies and initial singing rates. For each drug, relatively low and high doses were administered, based on similar experiments in previous studies (0.2 and 1 mg/kg for URB597 and 1 and 5 mg/kg for VDM11) (Manduca et al., 2014; Trezza and Vanderschuren, 2009, 2008a, 2008b). No significant effects of URB597 were found on either first song latencies (Figure 2A *left*; n = 12 birds, *p* = 1 and 0.47 for 0.2 mg/kg and 1 mg/kg, respectively) or initial singing rates (Figure 2A *right*; n = 12 birds, *p* = 0.21 and 0.76 for 0.2 mg/kg and 1 mg/kg, respectively). Similarly, no significant effects of VDM11 were observed for either first song latencies (Figure 2B *left*; n = 12 birds, *p* = 0.97 and 0.47 for 1 mg/kg and 5 mg/kg, respectively) or initial singing rates (Figure 2B *right*; n = 12 birds, *p* = 0.044 and 0.52 for 1 mg/kg and 5 mg/kg, respectively, significance level α was corrected from 0.05 to 0.0167).

## Discussion

Our results showing the marked increases in first song latencies by WIN administration and substantial reversal of those effects by SR administration suggest that endocannabinoid signaling through CB_1_ receptors is critically involved in the initiation of the first song after temporary singing suppression. Given that behavioral latencies have generally been used to quantify the levels of motivation for various behaviors including birdsong (Salamone and Correa, 2012; Volkow et al., 2017; Berke, 2018; Mohebi et al., 2019; Haakenson, Balthazart, and Ball 2020), these results demonstrate that endocannabinoids critically contribute to the regulation of intrinsic motivation for undirected singing. These findings greatly advance the understanding of the role of endocannabinoids in undirected singing. A previous study using the same drugs and a similar method examined the effects of these drugs on the amount of singing (the number of song bouts over a 90-min period) and demonstrated that the singing amount was decreased by WIN and partially reversed by SR (Soderstrom and Johnson, 2001). Since undirected song production has been suggested to act as internal reward (Riters and Stevenson, 2012), which may facilitate subsequent song production, the singing amount could reflect not only singing motivation, but also singing-associated reward. Thus, the previous study measuring only singing amounts does not demonstrate whether endocannabinoids are involved in intrinsic singing motivation, singing-associated reward, or both. Because the first song latency directly reflects intrinsic singing motivation independent of singing-associated reward (Kim et al., 2021), our results provide the first evidence, to our knowledge, of the significant role of endocannabinoids in intrinsic motivation for undirected singing in songbirds. The findings are consistent with those of previous studies in mammals showing that direct cannabinoid agonists, including WIN, affect the motivational aspects of both food- and drug-seeking behaviors (for review, see Parsons and Hurd, 2015).

The results of reduced initial singing rates following WIN administration also support the involvement of endocannabinoids in intrinsic singing motivation because reduced motivation for singing should result in reduced singing rates. However, these results do not eliminate the possibility that endocannabinoids are involved in singing-associated reward. As in the case of the singing amount described above, it is possible that WIN indirectly decreases initial singing rates by affecting singing-associated reward, in addition to directly affecting intrinsic singing motivation. Thus, endocannabinoids could play a role in singing-associated reward as well as in intrinsic singing motivation. In accordance with this idea, significant correlations between singing-associated reward and endocannabinoid-related gene expression have been found in songbirds (Hahn et al., 2017). This idea is also consistent with the roles of endocannabinoids in both motivational and pleasurable aspects of various behaviors in mammals (for review, see Sagheddu et al., 2015; Solinas et al., 2008).

We previously demonstrated that dopamine plays a critical role in regulating intrinsic motivation for undirected singing though D2 receptors (Kim et al., 2021). Because endocannabinoids modulate the dopamine system in mammals (for review, see Wenzel and Cheer, 2018), it is likely that endocannabinoids regulate intrinsic singing motivation by interacting with the dopamine system. In mammals, the interactions between endocannabinoids and the dopamine system vary across different brain areas (Wenzel and Cheer, 2018; Lovinger et al., 2022). For example, endocannabinoids disinhibit dopaminergic neurons in the midbrain by suppressing GABA releases from GABAergic interneurons, resulting in enhanced dopaminergic neuron activity and enhanced dopamine release from their axon terminals (Wang and Lupica, 2014). In contrast, endocannabinoids also have a function to decrease dopamine release via local actions in the striatum: dopamine release enhanced by glutamatergic inputs to the striatum is inhibited by the activation of CB_1_ cannabinoid receptors expressed on cortical glutamatergic terminals (Covey et al., 2017). Moreover, endocannabinoids differentially interact with dopamine D1 and D2 receptors (El Khoury et al., 2012), and these receptors are differentially involved in motivational processes (Olivetti et al., 2019; Volkow et al., 2017). In songbirds, it remains unclear how endocannabinoids interact with dopamine signaling to regulate singing motivation, although both dopamine-related signals and endocannabinoid-related signals are associated with the production of undirected song in many brain areas such as VTA, the medial preoptic area, and the periaqueductal gray, and the songbird basal ganglia nucleus Area X (Haakenson et al., 2020; Hahn et al., 2017; Heimovics et al., 2009, 2011; Kubikova et al., 2010; Merullo et al., 2016; Yanagihara and Hessler, 2006). Identifying the neural circuits and detailed mechanisms underlying the interaction between endocannabinoids and dopamine signaling will advance our understanding of how those neuromodulators regulate singing motivation.

In contrast with the direct cannabinoid agonist WIN, which binds to CB_1_ cannabinoid receptors, the indirect cannabinoid agonists URB597 and VDM11 increase cannabinoid binding to receptors by preventing the breakdown or reuptake of cannabinoids, respectively. These indirect cannabinoid agonists enhance social play in rats, an intrinsically motivated rewarding behavior (Manduca et al., 2014; Trezza and Vanderschuren, 2009, 2008a, 2008b). Such enhancing effects on social play are not consistent with our results of no significant effects of these drugs on undirected singing. This discrepancy could result from different mechanisms between birdsong and social play, and/or between animal species. In rodents, the effects of URB597 on social play depend on the age, strain, and behavioral context, and such effect patterns vary across different behaviors (Manduca et al., 2014). Given that songbirds have discrete neural circuits specialized for song learning and production (for review, see Mooney, 2009), it is likely that the mechanisms by which endocannabinoids regulate intrinsic singing motivation differ in many aspects from mechanisms of other voluntary behaviors in other animals. Nevertheless, the tractable nature of zebra finch songs and song control circuits will enable us to understand the detailed neurophysiological mechanisms underlying intrinsic motivation for complex learned motor behaviors. Moreover, endocannabinoids in songbirds are implicated, not only in singing motivation, but also in song development (Soderstrom and Gilbert, 2013; Soderstrom and Johnson, 2003), song recognition (Whitney et al., 2003; Hahn et al., 2019), and stress responses (Dickens et al., 2015), illustrating the importance of songbirds as a useful model system to study the roles of endocannabinoids in various biological functions.

## Data Availability

The original contributions presented in the study are included in the article/Supplementary Material, further inquiries can be directed to the corresponding author.
